# The m6A methyltransferase METTL16 inhibits the proliferation of pancreatic adenocarcinoma cancer cells *via* the p21 signaling pathway

**DOI:** 10.3389/fonc.2023.1138238

**Published:** 2023-04-25

**Authors:** Fuming Xie, Yao Zheng, Wen Fu, Bojing Chi, Xianxing Wang, Junfeng Zhang, Jianyou Gu, Jingyang Yin, Qiang Zhou, Shixiang Guo, Lei Cai, Jiali Yang, Songsong Liu, Huaizhi Wang

**Affiliations:** ^1^ University of Chinese Academy of Sciences (UCAS) Chongqing School, Chongqing Medical University, Chongqing, China; ^2^ Chongqing Institute of Green and Intelligent Technology, Chinese Academy of Sciences (CAS), Chongqing, China; ^3^ Chongqing School, University of Chinese Academy of Sciences (UCAS), Chongqing, China; ^4^ Institute of Hepatopancreatobiliary Surgery, Chongqing General Hospital, University of Chinese Academy of Sciences (UCAS Chongqing), Chongqing, China; ^5^ Chongqing Key Laboratory of Intelligent Medicine Engineering for Hepatopancreatobiliary Diseases, Chongqing General Hospital, Chongqing, China; ^6^ Savaid Medical School, University of Chinese Academy of Sciences (UCAS), Beijing, China; ^7^ Department of Hepatobiliary Surgery, Hainan Hospital of People’s Liberation Army of China (PLA) General Hospital, Sanya, China

**Keywords:** pancreatic adenocarcinoma, METTL16, m6A, p21, cell proliferation

## Abstract

**Background:**

Many studies have reported that N6-methyladenosine (m6A) modification plays a critical role in the epigenetic regulation of organisms and especially in the pathogenesis of malignant diseases. However, m6A research has mainly focused on methyltransferase activity mediated by METTL3, and few studies have focused on METTL16. The aim of this study was to investigate the mechanism of METTL16, which mediates m6A modification, and its role in pancreatic adenocarcinoma (PDAC) cell proliferation.

**Methods:**

Clinicopathologic and survival data were retrospectively collected from 175 PDAC patients from multiple clinical centers to detect the expression of METTL16. CCK-8, cell cycle, EdU and xenograft mouse model experiments were used to evaluate the proliferation effect of METTL16. Potential downstream pathways and mechanisms were explored via RNA sequencing, m6A sequencing, and bioinformatic analyses. Regulatory mechanisms were studied through methyltransferase inhibition, RIP, MeRIP‒qPCR assays.

**Results:**

We found that METTL16 expression was markedly downregulated in PDAC, and multivariate Cox regression analyses revealed that METTL16 was a protective factor for PDAC patients. We also demonstrated that METTL16 overexpression inhibited PDAC cell proliferation. Furthermore, we identified a METTL16-p21 signaling axis, with downregulation of METTL16 resulting in inhibition of CDKN1A (p21). Additionally, METTL16 silencing and overexpression experiments highlighted m6A modification alterations in PDAC.

**Conclusions:**

METTL16 plays a tumor-suppressive role and suppresses PDAC cell proliferation through the p21 pathway by mediating m6A modification. METTL16 may be a novel marker of PDAC carcinogenesis and target for the treatment of PDAC.

## Graphical abstract

Graphic Mechanism of METTL16 in PDAC.
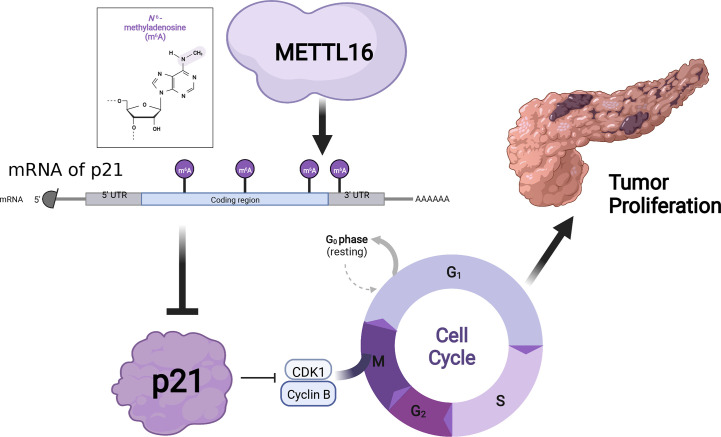

## 1 Introduction

Pancreatic cancer (PC) is a high-grade malignancy of the digestive system (1). After decades of development, the efficacies of surgery, chemotherapy and targeted drug treatment have improved slightly, yet the five-year survival rate is still less than 8% for PC (2, 3). Surgery is currently considered the only cure for PC. Nevertheless, because PC develops insidiously and quickly, the majority of patients are initially diagnosed with local invasion or distant metastasis and thus miss their chance for surgery, leading to a poor prognosis (4). The incidence of PC has been on the rise in recent years, and PC has become an important factor threatening people’s health. Elucidating the molecular mechanism of PC is the subject of current in-depth laboratory and clinical oncology studies and is essential for enhancing the efficacy of PC treatment.

N6-methyladenosine (m6A) methylation is one kind of RNA modification that occurs on adenines of RNAs, such as mRNAs and long noncoding RNAs (5, 6). m6A modification has been found in prokaryote, eukaryote and virus mRNAs and has a significant effect on the regulation of RNA-related biological processes (7, 8). Recently, several studies have shown that m6A plays a vital role in the regulation of biological processes, including stem cell differentiation, animal development, tumorigenesis and immunity (9, 10). m6A modification is a dynamic and reversible process that mainly involves three kinds of catalytic enzymes: writers (methyltransferases), erasers (demethylases) and readers (methylation-reading proteins) (11). m6A writers such as METTL3 (12) and METTL14 (13), which belong to the methyltransferase‐like (METTL) protein family, can catalyze adenosine methylation at the N6 position of RNA. METTL16 is another important protein in the METTL protein family, acting as an N6-adenosine methyltransferase (14). METTL16 contains the Rossmann-like fold of class I methyltransferases and uses S-adenosylmethionine (SAM) as a methyl donor with additional regulatory and RNA binding domains (15). Recent research has found that METTL16 can serve dual roles as both an m6A writer and reader to promote translation independent of m6A methylation (16). In diverse human cancers, including PC, METTL3 and METTL14 have been reported to play important roles (17–19). Whether METTL16 also plays a role in regulating proliferation by altering m6A modification in PC remains unclear.

Here, we discovered that the expression of METTL16 was decreased in human PC samples and that METTL16 might play a crucial role in cell proliferation. To clarify the role of METTL16 in PC, we further confirmed its effect on proliferation using RNA sequencing (RNA-seq) and MeRIP-seq analyses of PC cell lines and identified potential downstream CDKN1A-related mechanisms. The purpose of our study was to determine the function of the m6A methyltransferase METTL16 in PC *in vivo* and *in vitro*. The results suggest that METTL16 is a novel prognostic marker and potential therapeutic target for PC.

## 2 Materials and methods

### 2.1 Tissue specimens

This study was approved by the Chongqing General Hospital Ethics Committee. Cancerous and paracancerous fresh-frozen specimens were matched and obtained from patients pathologically diagnosed with pancreatic ductal adenocarcinoma (PDAC) at the Institute of Hepatopancreatobiliary Surgery of Chongqing General Hospital. All patients in this study signed informed consent forms. Follow-up of patients was performed by full-time staff every 3 months after surgery. All procedures met the guidelines of the Declaration of Helsinki.

### 2.2 Cell lines and culture

Five kinds of human PDAC cell lines (AsPC-1, BxPC-3, CFPAC-1, PANC-1, and SW1990; Chinese Academy of Sciences Cell Bank, Shanghai, China), were used in this study. BxPC-3 cells were maintained in RPMI 1640 medium (GIBCO, Invitrogen Inc., Carlsbad, CA, USA) supplemented with 10% fetal bovine serum (FBS) (GIBCO, Invitrogen Inc., Carlsbad, CA, USA), 100 U/ml penicillin and 100 U/ml streptomycin (GIBCO, Invitrogen Inc., Carlsbad, CA, USA). AsPC-1, PANC-1 and CFPAC-1 cells were cultured in DMEM with high glucose (GIBCO, Invitrogen Inc., Carlsbad, CA, USA) supplemented with 10% FBS (GIBCO, Invitrogen Inc., Carlsbad, CA, USA) and 1% antibiotics (GIBCO, Invitrogen Inc., Carlsbad, CA, USA). SW1990 cells were cultured in L15 medium (GIBCO, Invitrogen Inc., Carlsbad, CA, USA) supplemented with 10% FBS (GIBCO, Invitrogen Inc., Carlsbad, CA, USA) and 1% antibiotics (GIBCO, Invitrogen Inc., Carlsbad, CA, USA). All cells were incubated at 37°C in humidified air with 5% carbon dioxide (CO_2_).

### 2.3 m6A detection

Based on the manufacturer’s instructions, total m6A modifications in cells were detected using the m6A RNA Methylation Quantification Kit (Colorimetric, EpiQuik, USA). RNA high-binding solution was used to bind total RNA to wells. Capture and detection antibodies were used to detect m6A. Microplate readers were used to detect the signal at 450 nm. The OD was proportional to the m6A concentration.

### 2.4 RNA extraction and real-time PCR

Total RNA was isolated from PDAC tissues and cultured cells with an Ultrapure RNA kit (Cwbio, China) according to the manufacturer’s instructions. Reverse transcription was performed by a PrimeScript RT Reagent kit (Takara, Otsu, Japan) according to the manufacturer’s protocol. The SYBR^®^ Premix Ex Taq™ Kit (Takara, Otsu, Japan) was used for real-time PCR. Relative RNA expression levels were calculated by the 2-ΔΔCt method and normalized to GAPDH or β-actin levels. All primer pair sequences are presented in **Table S5**.

### 2.5 Cell transfection

CFPAC-1 and PANC-1 cells were used for METTL16 silencing experiments with short hairpin RNA (shRNA) purchased from RiboBio Co. (Guangzhou, China), and CFPAC-1 and SW1990 cells were used for METTL16 overexpression experiments with vectors (pGLV5/GFP/Puro, GeneChem, China). Both the shRNAs and vectors were transfected with Lipofectamine 3000 (Invitrogen, USA) according to the manufacturer’s instructions. Lentiviral infection was carried out according to the manufacturer’s protocol. Stable cells with overexpression or knockdown were obtained by selection with puromycin (5 μg/ml) for 2 weeks. All sequences are presented in **Table S5**.

### 2.6 Western blotting (WB) assay

RIPA lysis buffer (Sigma, USA) was used to lyse the cultured cells and tissues. The concentration was determined with the BCA Protein Assay Kit (Pierce antibodies, Thermo Fisher Scientific, Waltham, MA, USA). With a 4-20% gradient SDS-polyacrylamide gel (PAGE, GenScript, USA), proteins were separated by electrophoresis and transferred to a polyvinylidene difluoride (PVDF) membrane (Millipore, Billerica, MA, USA). The PVDF membranes were blocked with Rapid Blocking Buffer (Epizyme, Shanghai, China). The indicated primary antibody was incubated with the samples at 4°C overnight. The secondary antibody was incubated for an hour at room temperature, and an ECL chemiluminescence kit (GE, USA) was used for visualization. All antibody information is described in **Table S4**.

### 2.7 Immunohistochemistry (IHC) analysis

PDAC and paracancerous specimens were washed thrice with PBS buffer after dewaxing and hydration followed by antigen retrieval. Antigen retrieval was performed using the high-pressure method for 2–3 min in citrate buffer (pH 6.0). The specimens were brought to nearly room temperature, incubated in 3% H_2_O_2_ for 15 mins, and then blocked in 10% goat serum for 20 mins at room temperature. The sections were incubated with the primary antibody at 4°C overnight, and an anti-mouse/rabbit IHC detection kit (Dako, Glostrup, Denmark) was used to detect the immunohistochemical reaction. The staining intensity was calculated as follows: 0 (no staining), 1 (weak staining), 2 (moderate staining), and 3 (strong staining). The percentage of positively stained cells was calculated as follows: 0 (no staining), 1 (1–10% of cells with staining), 2 (10–50% of cells with staining), and 3 (more than 50% of cells with staining). The staining index (SI) was calculated by multiplying the staining intensity and percentage of positively stained cell values. PDAC specimens were categorized into lower expression (SI less than 4) and higher expression (SI more than 6) groups based on the SI.

### 2.8 Cell proliferation analysis

The proliferation ability of PC cells was detected by the Cell Counting Kit 8 (CCK-8) assay and the 5-ethynyl-2′-deoxyuridine (EdU) immunofluorescent assay. PC cells were seeded at 2 × 10^3^ per well in 96-well plates after transient siRNA transfection for 48 h. Every 24 hours, three wells per group were assessed with CCK-8 assay, and 10 μL of prepared solution was incubated for one hour at 37°C. The OD was measured at 450 nm by a microplate reader. The EdU assay (RiboBio, Guangzhou, China) was performed by seeding 2 × 10^4^ transfected cells per well in 24-well plates. The cells were then incubated with EdU buffer for two hours, followed by immunofluorescence staining. A detailed protocol was provided by the manufacturer.

### 2.9 Cell cycle analysis

Treated PC cells were collected and fixed in 75% pre‐chilled ethanol at 4°C overnight. After removing the ethanol, the cells were washed twice with phosphate buffer and incubated with RNase and propidium iodide (Beyotime, China). Cell cycle distribution was analyzed using a BD Accuri C6 flow cytometer. All the experiments were repeated independently in triplicate.

### 2.10 Animal experiments

Animal experiments complied with the Chongqing Medical University of Medicine Policy on the Care and Use of Laboratory Animals. Six-week-old female BALB/c nude mice were obtained from Peking University Animal Center (Beijing, China). These mice were randomized into experimental (N = 8 per group) and control groups (N = 8 per group). PC cells were resuspended in PBS (approximately 2 × 10^6^ cells/100 μL) and subcutaneously inoculated into the left armpit of each nude mouse. The tumor length and width of the mice were monitored weekly. All mice were sacrificed 5 weeks postinjection, and tumors were excised and weighed.

### 2.11 RNA-seq analysis

PANC-1 cells used for sequencing were stably transfected with si-METTL16 and control siRNA. Biological triplicates were set up for each experimental group. Total RNA was extracted from cells using an Ultrapure RNA kit (Cwbio, China). The quality and purity of total RNA extracted from the samples were analyzed by NanoDrop 2000 and Agilent Bioanalyzer 2100 measurements. RNA-seq was performed by SHBIO (Shanghai, China) with the Illumina HiSeq 2500 platform. Differential analyses between the si-METTL16 and control groups were performed by the R package limma.

### 2.12 m6A-RNA immunoprecipitation and MeRIP-seq analysis

MeRIP assays were performed using a Magna MeRIP kit (Merck Millipore, USA) according to the standard manual. The RNA was segmented and incubated with an anti-m6A antibody before immunoprecipitation. We detected the enrichment of m6A in RNA by high-throughput sequencing, constructed m6A-MeRIP RNA fragments with the Illumina NEBNext Ultra RNA Library Preparation kit, and sequenced the m6A-MeRIP RNA fragments using an Illumina HiSeq 2000. SHBIO (Shanghai, China) completed the library preparation. Sequencing reads were compared with the human genome build GRCh38.p13 through Bowtie2. m6A peaks were detected by the magnetic cell sorting method. Differential analyses between the input and MeRIP groups were performed by the R package limma.

### 2.13 Differential gene enrichment analysis

#### 2.13.1 Gene Set Enrichment Analysis (GSEA)

GSEA was performed using GSEA 3.0 software. The gene sets mentioned in the article were downloaded using MSigDB (http://software.broadinstitute.org/gsea/index.jsp). The number of permutations was set to 1,000.

#### 2.13.2 Pathway enrichment analysis

Pathway enrichment analysis of the key DEGs in our research was performed using Gene Ontology (GO) and Kyoto Encyclopedia of Genes and Genomes (KEGG) databases.

### 2.14 Statistical analysis

Normally distributed continuous data are presented as the mean ± standard deviation (SD) of three independent experiments and were compared with Student’s t test. A logistic regression model was used to analyze dichotomous variables. The Cox proportional hazards regression model was used to identify independent prognostic factors. The Kaplan−Meier method was used to analyze the survival data. Correlations were examined using Pearson coefficients. p<0.05 was considered statistically significant. All the figures were generated by GraphPad Prism software version 8.1. Statistical analyses were performed using SPSS 26.0 statistical software for Windows (IBM, Armonk, NY, USA).

## 3 Results

### 3.1 METTL16 is expressed at low levels in PDAC and suggests a poor prognosis

**Figure 1:**
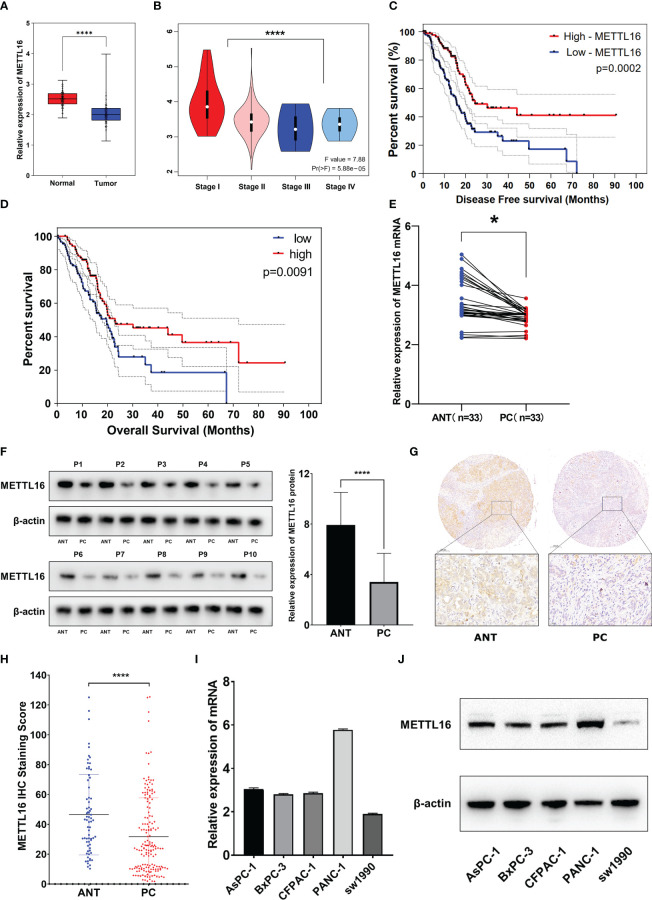
METTL16 is downregulated in PDAC tissues, and downregulation of METTL16 suggests poor prognosis. **(A)** The mRNA level of METTL16 in normal tissues and PC tissues in data downloaded from The Cancer Genome Atlas (TCGA) and GTEx database (p < 0.001). **(B)** mRNA level of METTL16 in different PC stages (I‐IV) according to data downloaded from the TCGA database (p < 0.05). Cox regression multivariate analysis showed that the expression level of METTL16 was significantly positively correlated with both the DFS **(C)** and OS**(D)** from TCGA. **(E)** METTL16 expression was assessed by qRT−PCR in paired PC tissues and ANTs (N = 33). **(F)** Western blotting was performed to detect the protein level of METTL16 in 10 PC tissues and paired ANTs. **(G)** METTL16 expression was assessed by IHC in PC tissue microarrays and two independent clinical (N = 133) and ANT (N = 133) sample cohorts. **(H)** The METTL16 staining score in adjacent non-cancerous tissue was significantly higher than that in PC tissues. **(I)** mRNA levels of METTL16 in 5 PC cell lines (AsPC-1, BxPC-3, CFPAC-1, PANC-1, and SW1990). **(J)** Protein level of METTL16 in 5 PC cell lines. * means p<0.05; **** means p<0.0001.

We found that among the METTL family members, METTL16 is a vital gene for the survival of the vast majority of cancer cells, implying its functional importance in cancer. To explore the expression of METTL16 in PDAC, we compared the expression profiles between PDAC tissues and adjacent non-cancerous tissue with an mRNA expression dataset from The Cancer Genome Atlas (TCGA) and GTEx (https://xenabrowser.net/datapages/). The results showed that the level of METTL16 in PDAC tissues was significantly lower than that in paired paracancerous nontumor tissues (**Figure 1A**), and the mRNA expression level of METTL16 was relatively higher in patients with a relatively low PC stage (I-II) than in those with a high PC stage (III-IV) (**Figure 1B**). Subsequently, by analyzing METTL16 expression levels and prognosis in patients with PDAC, we found that a high level of METTL16 was associated with significantly increased disease-free survival (DFS) by Kaplan−Meier analysis (**Figure 1C**). DFS showed a similar trend, with a low level of METTL16 being correlated with poor overall survival (OS) (**Figure 1D**).

The expression of METTL16 in 33 cases of PDAC and paired adjacent non-cancerous tissue (ANT) was visualized by real-time qPCR and WB. Consistent with the results of the TCGA and GTEx analyses, our results showed that the mRNA level of METTL16 was decreased in PC tissues, while it was increased in paired ANT (**Figure 1E**), which also showed increased protein levels of METTL16 (**Figure 1F**). To verify the METTL16 expression level in PDAC patients, tissue microarrays from a total of 133 PC patients from two clinical surgical centers were analyzed by IHC. Our data demonstrated that the SI Staining score of METTL16 in PDAC tissues was significantly decreased compared with that in paired ANT (**Figures 1G, H**). We determined the relationships between clinicopathological variables and the level of METTL16, and the results are shown in **Table 1**. Interestingly, patients with larger tumors tended to show lower levels of METTL16 in tumors (**Table 1**). In addition, logistic regression showed that METTL16 expression in PC tissues was closely related to the size of the tumor (OR, 0.253, p< 0.001), but depth of tumor invasion (OR, 1.186, p= 0.723) and degree of metastasis (OR, 0.89, p= 0.816) did not appear to be directly related to METTL16 expression (**Table 2**). Similarly, in the TCGA PC data analysis, we also found that METTL16 expression was correlated with the size of PDAC tumors (**Table S1**). We detected the mRNA level (**Figure 1I**) and protein level (**Figure 1J**) of METTL16 in five PC cell lines and found that the levels were higher in PANC-1 cells and lower in SW1990 cells.

**Table 1:** Associations of METTL16 expression with clinical parameters in pancreatic cancer.

Characteristic	No.	METTL16 expression		P-value
		Low(N=72)	High(N=83)	
**Age (year)**		66.1	63.3	
<=60	48	18 (25.0)	30 (36.1)	0.186
>60	107	54 (75.0)	53 (63.9)	
**Gender**				0.194
Female	70	28 (38.9)	42 (50.6)	
Male	85	44 (61.1)	41 (49.4)	
**Tumor size**				<0.001*
<= 4cm	101	35 (48.6)	66 (79.5)	
> 4cm	54	37 (51.4)	17 (20.5)	
**Depth of invasion**				0.376
T1+T2	116	51 (70.8)	65 (78.3)	
T3+T4	39	21 (29.2)	18 (21.7)	
**Phase**				<0.001*
I-II	44	9 (12.5)	35 (42.2)	
III-IV	111	63 (87.5)	48 (57.8)	
**Lymph node metastasis**				<0.001*
N0	46	10 (13.9)	36 (43.4)	
N+	109	62 (86.1)	47 (56.6)	

The bold values in the first column mean Characteristic title. The bold values in the p-value column mean the result statistically significant. The symbol * means the result statistically significant.

**Table 2:** Multivariate logistic regression analysis.

Characteristic	OR	P-value	95% CI
**Age**	2.641	0.023	1.145-6.088
**Gender**	0.695	0.348	0.325-1.486
**Tumor Size**	4.598	<0.001*	1.998-10.58
**Depth of invasion**	1.339	0.531	0.538-3.335
**Phase**	11.917	0.117	0.538-263.98
**Lymph node metastasis**	0.636	0.765	0.032-12.446

The bold values in the first column mean Characteristic title. The bold values in the p-value column mean the result statistically significant. The symbol * means the result statistically significant.

### 3.2 Downregulation of METTL16 promotes PC cell proliferation by restraining cells in the G1 phase

**Figure 2:**
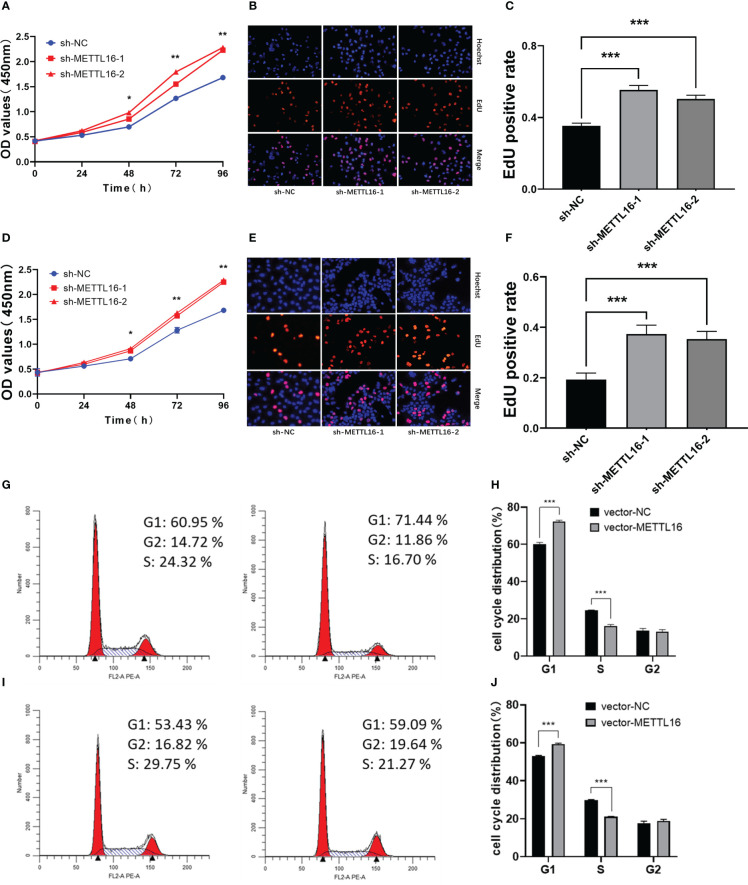
Downregulation of METTL16 promotes PC cell proliferation by restraining cells in the G1 phase. CCK-8 assay to assess the effect of METTL16 on PANC-1 cell **(A)** and CFPAC-1 cell **(D)** growth. EdU assays were used to assess cell proliferation. The histogram shows the proliferation rates of transfected cells in the corresponding groups: PANC-1 **(B, C)**; CFPAC-1 **(E, F)**. Flow cytometry assays showed that the overexpression of METTL16 significantly increased the proportion of CFPAC-1 **(G, H)** and SW1990 **(I, J)** cells in the G1 phase and decreased the proportion of cells in the S phase. * means p<0.05; ** means p<0.01; *** means p<0.001.

According to the expression levels of METTL16 in different PC cell lines, PANC-1 and CFPAC-1 cells were selected for the subsequent knockdown experiment, while SW1990 and CFPAC-1 cells were selected for the subsequent overexpression experiment. shRNA was applied to knock down METTL16, while a vector was used for overexpression in PC cell lines. The METTL16 knockdown efficiency is shown in **Figures S1A, B**, and the METTL16 overexpression efficiency is shown in **Figures S1C, D**. We found that silencing METTL16 significantly promoted cell growth using CCK8 and EdU assays in PANC-1 (**Figures 2A–C**) and CFPAC-1 (**Figures 2D–F**) cells, respectively. The results from flow cytometry demonstrated that PANC-1 (**Figures 2G, H**) and CFPAC-1 cells (**Figures 2I, J**) with METTL16 overexpression were arrested in the G1 phase. The above results suggested that METTL16 was able to inhibit PC cell proliferation by delaying the G1 phase transition.

### 3.3 METTL16 inhibits tumor growth in mice

**Figure 3:**
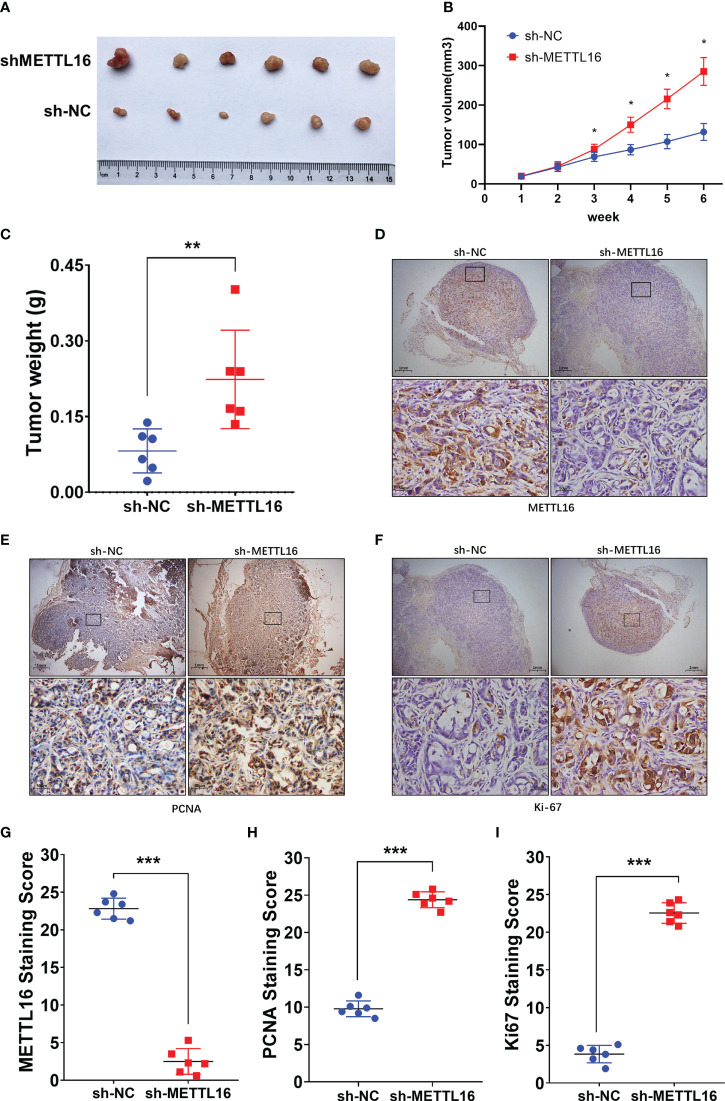
METTL16 inhibits tumor growth in mice. **(A)** Nude mice were subcutaneously implanted with sh-NC or sh-METTL16 PC cells, and subcutaneous tumor nodules formed in the two groups of mice. **(B)** The weekly tumor volumes of the METTL16-knockdown and control groups are presented in the chart. **(C)** The mean tumor weights of the METTL16-knockdown and control groups 6 weeks after inoculation are presented. Two groups of tumor specimens were subjected to immunohistochemical detection of METTL16 **(D, G)**, PCNA **(E, H)** and Ki67 **(F, I)**. * means p<0.05; ** means p<0.01; *** means p<0.001.

To verify the vital role of METTL16 in PC cell proliferation *in vivo*, we established a xenograft model in nude mice by subcutaneously injecting PANC-1 cells with stable knockdown of METTL16 and control PANC-1 cells (**Figure 3A**). The volume (**Figure 3B**) and weight (**Figure 3C**) of subcutaneous xenografts were markedly increased in the METTL16-silenced groups compared with the control group, implying a crucial role of METTL16 in PC cell growth *in vivo*. IHC staining showed that the protein level of METTL16 was significantly decreased in METTL16-silenced cells compared with control cells (**Figures 3D, G**). Moreover, METTL16 knockdown led to increased expression of Ki-67 (**Figures 3E, H**) and PCNA (**Figures 3F, I**), two proteins correlated with cancer cell proliferation, according to IHC staining, which was in line with the results of mouse tumor growth. Overall, the above data demonstrated that METTL16 exerted a great influence on PC cell proliferation *in vivo*.

### 3.4 RNA-seq analysis revealed the underlying mechanisms by which METTL16 affects PC cell proliferation

**Figure 4:**
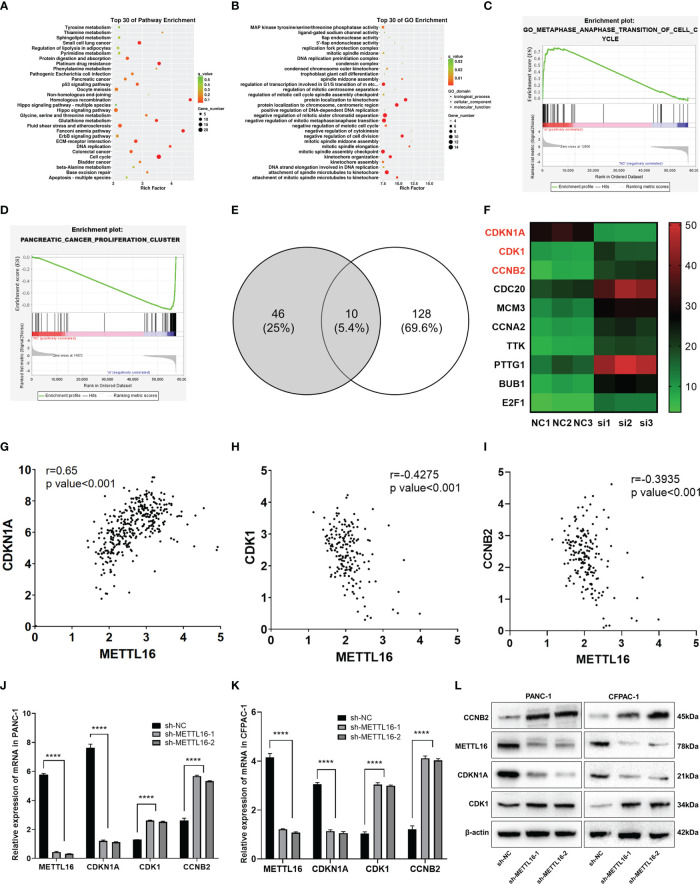
RNA-seq analysis revealed the underlying mechanisms by which METTL16 affects PC cell proliferation. **(A, B)** KEGG and GO analyses of METTL16-knockdown PANC-1 cells. **(C, D)** Gene sets related to proliferation and cell cycle signaling pathways were identified through GSEA. **(E)** The Venn diagram presents 10 common target genes. **(F)** Heatmaps of the 10 selected target genes. Analysis of the correlation of METTL16 expression with CDKN1A **(G)**, CDK1 **(H)**, and CCNB2 **(I)** expression was performed with TCGA data. RT−qPCR analysis of the mRNA expression of METTL16, CDKN1A, CDK1 and CCNB2 in PANC-1 **(J)** and CFPAC-1 **(K)** cells was performed. **(L)** Western blotting analysis was performed to detect the expression of proteins associated with METTL16, CDKN1A, CDK1 and CCNB2 in PANC-1 (left) and CFPAC-1 (right) cells. **** means p<0.0001.

To further investigate the downstream molecular mechanisms by which METTL16 affects PC cell proliferation, RNA-seq analysis was performed. First, the transcriptomics data of METTL16-silenced and control PANC-1 cells were analyzed, revealing differential gene expression and pathways related to METTL16 (**Figure S2A, B**). Clustering analysis (**Figures S2C, D**) suggested that METTL16 significantly affects the expression of key PC-related genes. Kyoto Encyclopedia of Genes and Genomes (KEGG) and Gene Ontology (GO) analyses based on differentially expressed genes revealed the top 30 enriched biological pathways among METTL16-silenced versus control cells (**Figures 4A, B**). We then conducted gene set enrichment analysis (GSEA, Broad Institute) based on the RNA-seq results, which revealed that silencing of METTL16 was strongly correlated with gene sets associated with cell cycle (**Figure 4C**) and PC cell proliferation (**Figure 4D**) signaling pathways. Additionally, we performed integrated analysis of PANC-1 cell RNA-seq data and identified the enriched cell cycle- and proliferation-related genes with the most significant regulatory changes. After combining the two subsets, we identified 10 key genes through Venn diagram analysis (**Figure 4E**), and several important genes that are highly associated with tumors were found, indicating that key downstream regulatory networks in PC were closely associated with METTL16 (**Figure 4F**). To further examine the roles these genes play, we used TCGA PDAC and GTEx pancreas data for correlation analysis and subsequently found that several genes, including CDKN1A (p21) (**Figure 4G**), CDK1 (**Figure 4H**) and CCNB2 (**Figure 4I**), which are involved in the G1 phase transition, were highly related to METTL16 in clinical specimens. To illuminate the mechanism by which METTL16 regulates G1 phase arrest, we determined the mRNA expression of CDKN1A, CDK1 and CCNB2. We found that among METTL16-silenced PANC-1 and CFPAC-1 cells, CDKN1A expression was significantly reduced, while CDK1 and CCNB2 expression was increased (**Figures 4J, K**). Similarly, the WB results revealed that the protein level of CDKN1A was significantly decreased and that CDK1 and CCNB2 were upregulated in PC cells after METTL16 silencing (**Figure 4L**). In summary, the RNA-seq analysis showed the essential regulatory role of METTL16 in PC. METTL16 might affect tumorigenesis and development by regulating vital molecules in the p21 signaling pathway.

### 3.5 MeRIP-seq analysis revealed that METTL16 activates CDKN1A *via* m6A-related mechanisms

**Figure 5:**
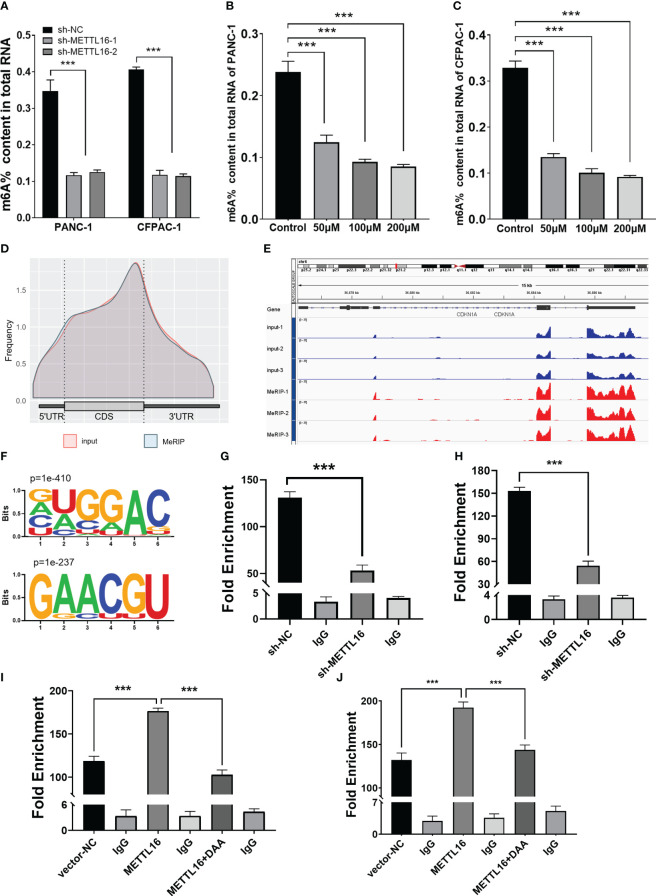
MeRIP-seq analysis revealed that METTL16 activates CDKN1A *via* m6A-related mechanisms. **(A)** Quantitative RNA methylation analysis based on a standard curve was used to detect the overall content of m6A in sh-NC and sh-METTL16 PANC-1 and CFPAC-1 cells. The global level of m6A methylation in PANC-1 **(B)** and CFPAC-1 **(C)** cells treated with different concentrations of DAA (0 µmol/L, 100 µmol/L, 200 µmol/L) for 24 h are shown. **(D)** Metagene plot of the m6A peak distribution in mRNA. **(E)** IGV analysis showed the binding peaks of the target gene (CDKN1A) in the genome. **(F)** Global profiling of m6A in PANC-1 cells with the most interesting sequence motifs of m6A peaks indicated. MeRIP-qPCR assays with an anti-m6A antibody or IgG were performed to verify the enrichment of CDKN1A mediated by METTL16 at m6A-related peaks of interest. The m6A modification of CDKN1A was increased upon overexpression of METTL16 **(I, J)**, while it was depleted upon knockdown of METTL16 **(G, H)** or addition of DAA **(I, J)** in both PANC-1 and CFPAC-1 cells. *** means p<0.001.

METTL16 acts as a methyltransferase, and we sought to further demonstrate whether METTL16 regulates CDKN1A expression by mediating its mRNA N6-methylation. First, we found that total m6A modification levels in PC cells were significantly reduced after METTL16 was silenced (**Figure 5A**). In addition, we used the methyltransferase inhibitor 3-Deazaadenosine (DAA) to treat PANC-1 (**Figure 5B**) and CFPAC-1 (**Figure 5C**) cells; the results showed that the m6A modification level decreased significantly after DAA reached a certain concentration. To clarify the key role of METTL16 in m6A modification, we performed MeRIP-seq analysis to map m6A modifications in PANC-1 cells to identify the base sites of downstream molecules regulated by METTL16 (**Figure 5D**). Importantly, through MeRIP-seq in PANC-1 cells, an m6A peak was found in the stop codon of CDKN1A mRNA (**Figure 5E**), which suggested that the CDKN1A transcript might be a direct target of m6A modification. m6A modification mostly occurs in the RRACH (R=G or A, H=A, C or U) consensus sequence. Our MeRIP-seq data demonstrated that the GUGGAC and GAACGU motifs were highly enriched (**Figure 5F**). Interestingly, these two motifs are also present in the exons of CDKN1A. The MeRIP-seq results were validated by MeRIP-qPCR. Compared with the IgG rabbit control antibody, the m6A-specific antibody markedly reduced the level of CDKN1A mRNA upon METTL16 knockdown in PANC-1 (**Figure 5G**) and CFPAC-1 cells (**Figure 5H**), while the m6A-specific antibody significantly increased the CDKN1A mRNA level upon METTL16 overexpression, and adding DAA reversed this effect (**Figures 5I, J**).

### 3.6 METTL16 regulates the m6A modification of CDKN1A to inhibit PC cell proliferation

**Figure 6:**
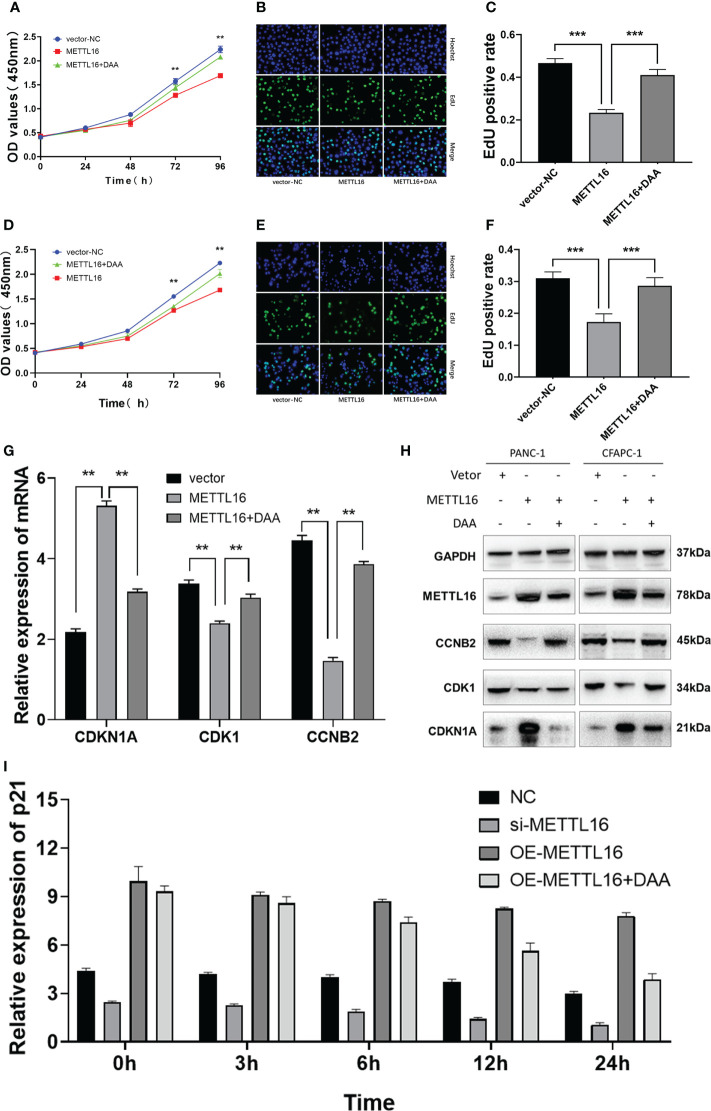
METTL16 regulates the m6A modification of CDKN1A to inhibit the proliferation of PC. Overexpression of METTL16 significantly inhibited cell growth according to CCK8 cell viability and EdU assays, and DAA administration rescued the proliferative capacity of both SW1990 **(A–C)** and CFPAC-1 cells **(D–F)**. **(G)** RT−qPCR showed that METTL16 overexpression modulated the mRNA levels of CDKN1A, CDK1 and CCNB2, while DAA administration restored their levels. **(H)** Western blotting analysis was performed to detect CDKN1A, CDK1 and CCNB2 expression in SW1990 and CFPAC-1 cells treated with METTL16-vector and DAA. **(I)** The stability of RNA level of CDKN1A was significantly decreased after the addition of DAA. ** means p value < 0.01, *** means p value < 0.001.

We sought to further characterize how METTL16 induces anticancer effects through CDKN1A in PC. We found that overexpression of METTL16 significantly inhibited cell growth through CCK8 and EdU assays in SW1990 (**Figures 6A–C**) and CFPAC-1 cells (**Figures 6D–F**), and DAA rescued cell proliferative capacity. As expected, we found that in METTL16-overexpressing SW1990 and CFPAC-1 cells, the mRNA level of CDKN1A was significantly increased, while the levels of CDK1 and CCNB2 were decreased. In addition, DAA rescued the levels of these mRNAs (**Figure 6G**). Similarly, the WB results showed that the protein level of CDKN1A was significantly increased while those of CDK1 and CCNB2 were reduced in PC cells after transfection of the METTL16 vector, and the protein level changes could be reversed by DAA administration (**Figure 6H**). Thus, our data suggest that METTL16 regulates the m6A modification of CDKN1A to inhibit the proliferation of PC cells. Moreover, the expression level of CDKN1A in PANC-1 cells was significantly increased after overexpressed METTL16, and it was affected by actinomycin D (**Figure 6I**). This further demonstrated that METTL16 could increase CDKN1A expression by regulating its RNA stability.

## 4 Discussion

More than one hundred different types of posttranscriptional modifications have been found on RNA, and all known types of RNA possess distinct chemical modifications, among which m6A modification is the most abundant and the most studied (9, 20). Recently developed, antibody-based, high-throughput sequencing technologies have enabled researchers to precisely map the exact sites of m6A modification and further understand its biological roles (21, 22). Although m6A modification has been revealed to regulate mRNA decay (23), translation (24, 25) and other processes (26) and to play an important role in regulating tumor cell proliferation signals (27), resisting apoptosis (28), and inducing invasion and metastasis (29) and is expected to become a target for cancer therapy, cancer-related studies on the regulatory mechanism of m6A modification are still limited.

At present, most studies on methyltransferase-related m6A modification focus on METTL3 and METTL14. Both METTL3 and METTL14 contain an S-adenosyl methionine-binding motif (30). These proteins colocalize in nuclear speckles, form a heterodimer, and catalyze the covalent transfer of a methyl group to adenine with the assistance of WTAP (13, 31). In addition, METTL16, as the second m6A methyltransferase identified, has known substrates, including U6 snRNA and human MAT2A mRNA, which encodes the SAM synthetase (14, 32). Previous literature has reported that the m6A-modified region of METTL16 is different from that of METTL3 (33). Therefore, in our study, we conducted RNA-seq after METTL3 knockdown in PANC-1 cells (**Figure S3A, B**) and found that most of the differentially expressed genes were different from those after METTL16 silencing. Furthermore, there was a substantial difference in the enriched genes of PANC-1 cells after METTL16 knockdown (**Figure S3C, D**). In addition, we compared the top 250 genes with the most significant differences between the two groups and revealed that only 11 genes had a similar variation tendency (**Figure S3E**). Furthermore, unlike METTL3 or METTL14, which are mostly located in the nucleus, the WB analysis revealed that METTL16 was localized in both the nucleus and cytosol of PC cells (**Figure S4A**). As reported previously, m6A modifications are mainly installed with transcription in the nucleus (14, 34). Thus, in addition to catalyzing the formation of m6A, METTL16 probably has additional functions, particularly in the cytosol (16). Therefore, in addition to m6A modification, more attention needs to be paid to the cytoplasmic regulation of METTL16. Interestingly, when METTL3 was knocked down, the expression levels of CDKN1A, CKD1 and CCNB2 did not change significantly (**Figure S4B**). Although METTL3 and METTL16 are homologues, their downstream regulatory networks differ greatly.

In gastric cancer, researchers found that METTL16 is highly expressed in cancer tissues, and METTL16-mediated m6A methylation promotes GC cell proliferation by enhancing cyclin D1 expression (35). The regulatory effects of METTL16 on malignant tumors (16) such as breast cancer (36) and liver cancer (37) have also been reported. However, in our study, we revealed for the first time that the novel m6A writer METTL16 was significantly decreased in PC cells and played a tumor suppressor role during human pancreatic carcinogenesis and progression. We verified the key function of METTL16 in inhibiting PC cell proliferation using lentiviral-based gene silencing and vector-based gene overexpression systems both *in vitro* and *in vivo*. Moreover, we found that the addition of the p21 inhibitor UC2288 could rescue the phenotype of overexpressing METTL16 (**Figure S5**). The above results suggest that the expression level and functional roles of METTL16 in different tumors vary. Therefore, our study on METTL16-mediated m6A regulation of tumor progression is novel and of great significance.

Interestingly, we discovered a novel regulatory pathway of p21 in which METTL16 mediates the m6A modification of CDKN1A (p21) to regulate the downstream genes CDK1/CCNB1, thereby promoting the proliferation of PC cells. Cyclin-dependent kinase inhibitor 1A (CDKN1A), a member of the Clp family, is a cyclin-dependent kinase inhibitor downstream of p53 and is also known as p21 because of its molecular weight of 21 kD. The p21 protein is the cell cycle suppressor with the most extensive kinase-suppressing activity known at present. p21 inhibits the activity of cyclin D1-CDK4 and cyclin E-CDK2, preventing the phosphorylation of Rb protein and the release of E2F, which leads to cell cycle stagnation in the G1 phase and the inhibition of DNA replication (38). Previous studies have reported that p21 is closely related to KRAS mutation (39), cancer cell migration, invasion, proliferation, and cell cycle and the epithelial-mesenchymal transition of PC cells (40). As CDKN1A (p21) is a hub gene in cancer regulation, the modification of CDKN1A m6A is worthy of study. In liver cancer (41), esophageal cancer (42), lung cancer (43), breast cancer (44), cervical cancer (45) and acute leukemia (46), it has been reported that upstream molecules can be modified by m6A and play an important role through the p21 pathway. However, few studies have investigated the function of m6A modification of CDKN1A (p21) at the transcriptional level, and this is the focus of our research group.

Previous studies have found that METTL16 has two verified modification sequences that are different from those of METTL3 (33, 47), which seems to be inconsistent with our results. As such, we cannot definitively say that CDKN1A mRNA is a direct substrate of METTL16-mediated m6A modification, and it is unclear whether METTL16 can splice CDKN1A pre-mRNA or regulate the translation of CDKN1A. It remains to be determined whether METTL16 acts as a writer to modify CDKN1A mRNA with m6A and whether there is a recognition protein or reader that binds to it and performs the corresponding biological function.

In summary, our study demonstrates that the regulation of METTL16 and its m6A modifications inhibit pancreatic carcinogenesis by regulating the expression and stability of critical tumor suppressor genes at the posttranscriptional level. These results aid elucidation of the specific molecular mechanisms related to METTL16 and its modification of m6A and the potential diagnostic and therapeutic value of these factors in PC.

## Data availability statement

The original data presented in the study are included in the article and Supplementary Material. The RNA seq data have been deposited in the GEO database (GSE226518). Further inquiries can be required from the corresponding authors.

## Ethics statement

The animal study was reviewed and approved by the Animal Experiment Ethics Committee of Chongqing Medical University. Written informed consent was obtained from the individual(s) for the publication of any potentially identifiable images or data included in this article.

## Author contributions

Conception and design: FX, SL, HW; Development of methodology: FX, SL, YZ, XW; Acquisition of data (provided animals, acquired and managed patients, provided facilities, etc.): FX, YZ, JLY, WF, BC, JG, QZ; Analysis and interpretation of data (e.g., statistical analysis, biostatistics, computational analysis): FX, WF, BC, JYY, JG, and JZ; Writing, review, and/or revision of the manuscript: FX, SL, YZ, HW; Study supervision: LC, SG, HW. All authors contributed to the article and approved the submitted version.
