# Corrigendum: The m6A methyltransferase METTL16 inhibits the proliferation of pancreatic adenocarcinoma cancer cells via the p21 signaling pathway

**DOI:** 10.3389/fonc.2023.1218781

**Published:** 2023-05-19

**Authors:** Fuming Xie, Yao Zheng, Wen Fu, Bojing Chi, Xianxing Wang, Junfeng Zhang, Jianyou Gu, Jingyang Yin, Qiang Zhou, Shixiang Guo, Lei Cai, Jiali Yang, Songsong Liu, Huaizhi Wang

**Affiliations:** ^1^ University of Chinese Academy of Sciences (UCAS) Chongqing School, Chongqing Medical University, Chongqing, China; ^2^ Chongqing Institute of Green and Intelligent Technology, Chinese Academy of Sciences (CAS), Chongqing, China; ^3^ Chongqing School, University of Chinese Academy of Sciences (UCAS), Chongqing, China; ^4^ Institute of Hepatopancreatobiliary Surgery, Chongqing General Hospital, University of Chinese Academy of Sciences (UCAS Chongqing), Chongqing, China; ^5^ Chongqing Key Laboratory of Intelligent Medicine Engineering for Hepatopancreatobiliary Diseases, Chongqing General Hospital, Chongqing, China; ^6^ Savaid Medical School, University of Chinese Academy of Sciences (UCAS), Beijing, China; ^7^ Department of Hepatobiliary Surgery, Hainan Hospital of People’s Liberation Army of China (PLA) General Hospital, Sanya, China

**Keywords:** pancreatic adenocarcinoma, METTL16, m6A, p21, cell proliferation

## Error in Figure/Table

In the published article, there was an error in **Figures 3D, F** as published. The incorrect images were included. The corrected **Figures 3D, F** and its caption appear below. 

**Figure 3:**
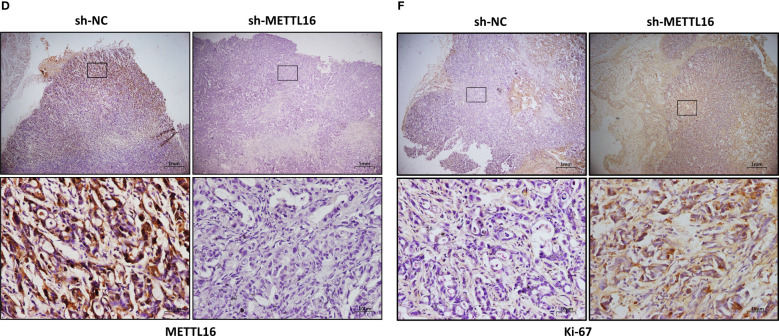
METTL16 inhibits tumor growth in mice. **(A)** Nude mice were subcutaneously implanted with sh-NC or sh-METTL16 PC cells, and subcutaneous tumor nodules formed in the two groups of mice. **(B)** The weekly tumor volumes of the METTL16-knockdown and control groups are presented in the chart. **(C)** The mean tumor weights of the METTL16-knockdown and control groups 6 weeks after inoculation are presented. Two groups of tumor specimens were subjected to immunohistochemical detection of METTL16 **(D, G)**, PCNA **(E, H)** and Ki67 **(F, I)**. * means p<0.05; ** means p<0.01; *** means p<0.001.

The authors apologize for this error and state that this does not change the scientific conclusions of the article in any way. The original article has been updated.

