# Supplementary figures and images for: The m6A methyltransferase METTL16 inhibits the proliferation of pancreatic adenocarcinoma cancer cells via the p21 signaling pathway
Source: Front Oncol. 2023 Apr 25;13:1138238. doi: 10.3389/fonc.2023.1138238 (PMC10166879; doi:10.3389/fonc.2023.1138238)

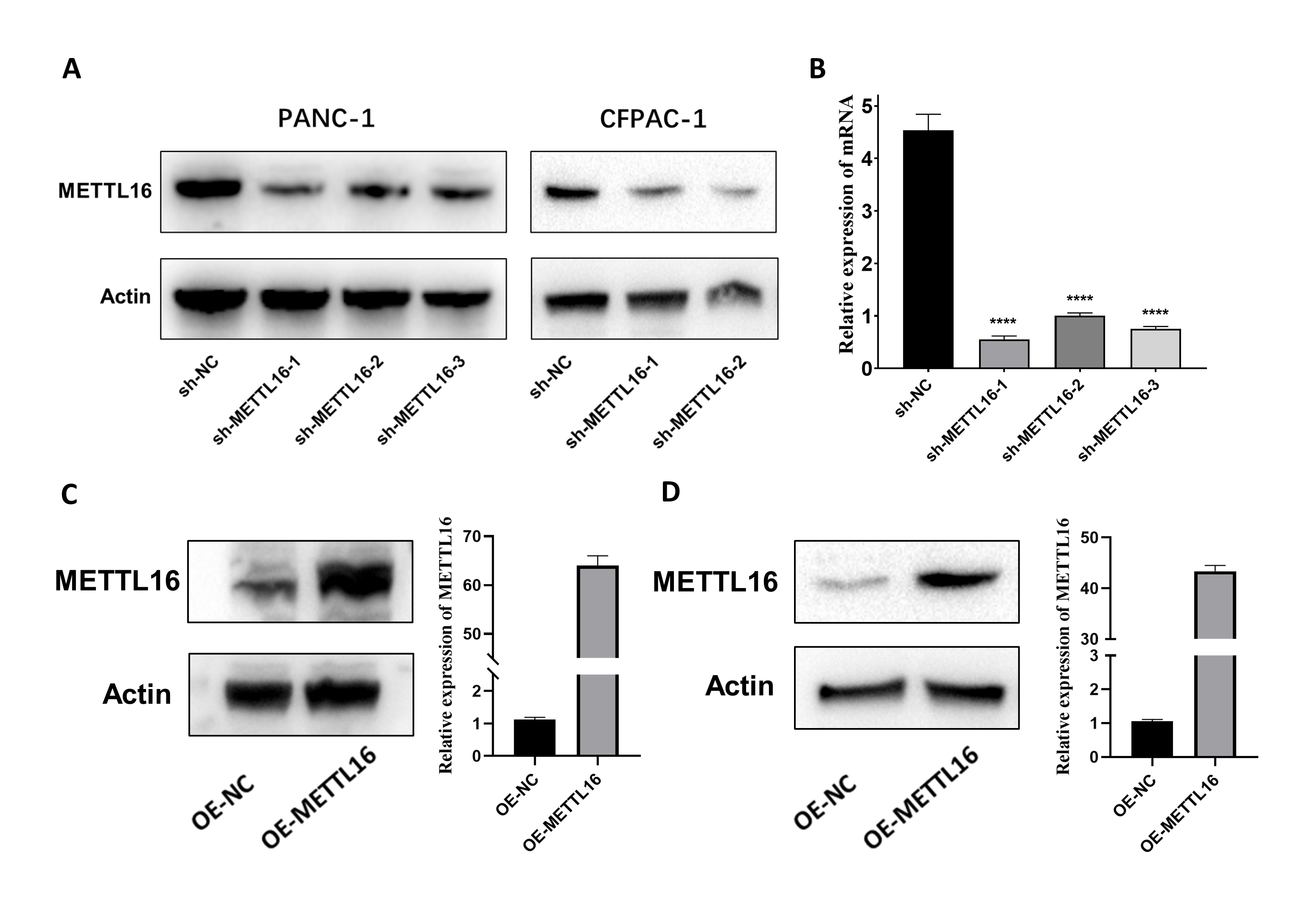

Supplement: Supplementary file 1 [file Image_1.tif]

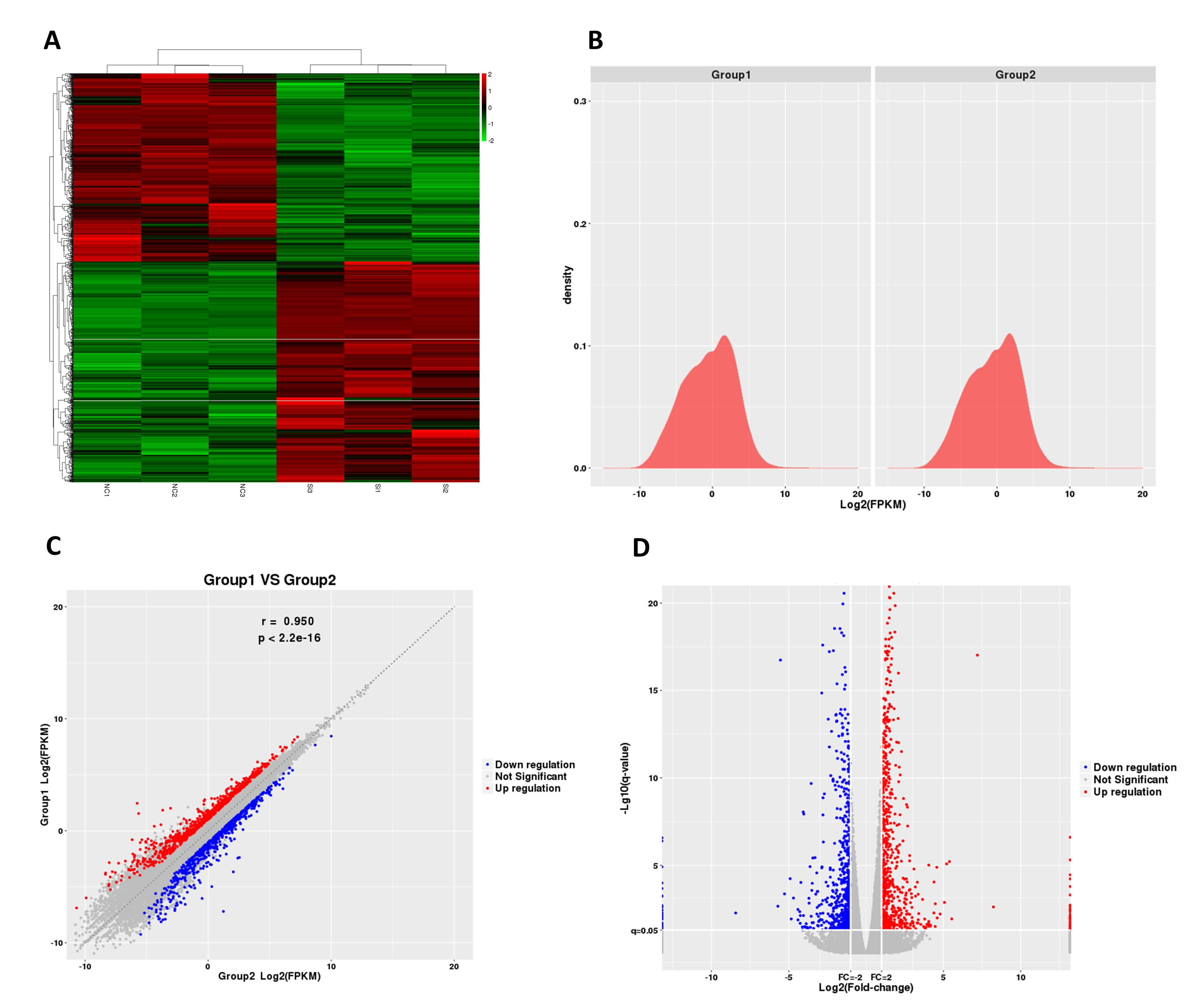

Supplement: Supplementary file 2 [file Image_2.tif]

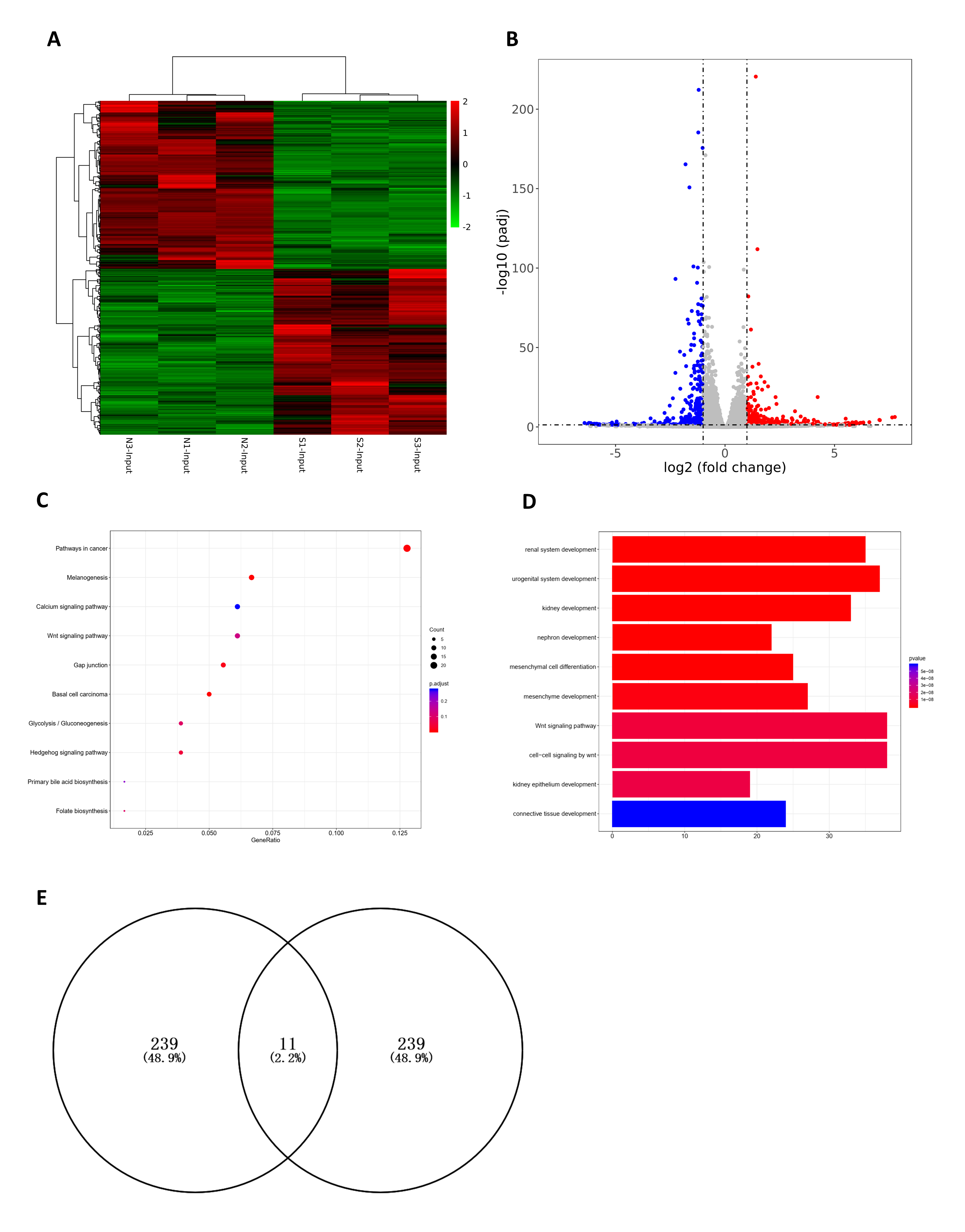

Supplement: Supplementary file 3 [file Image_3.tif]

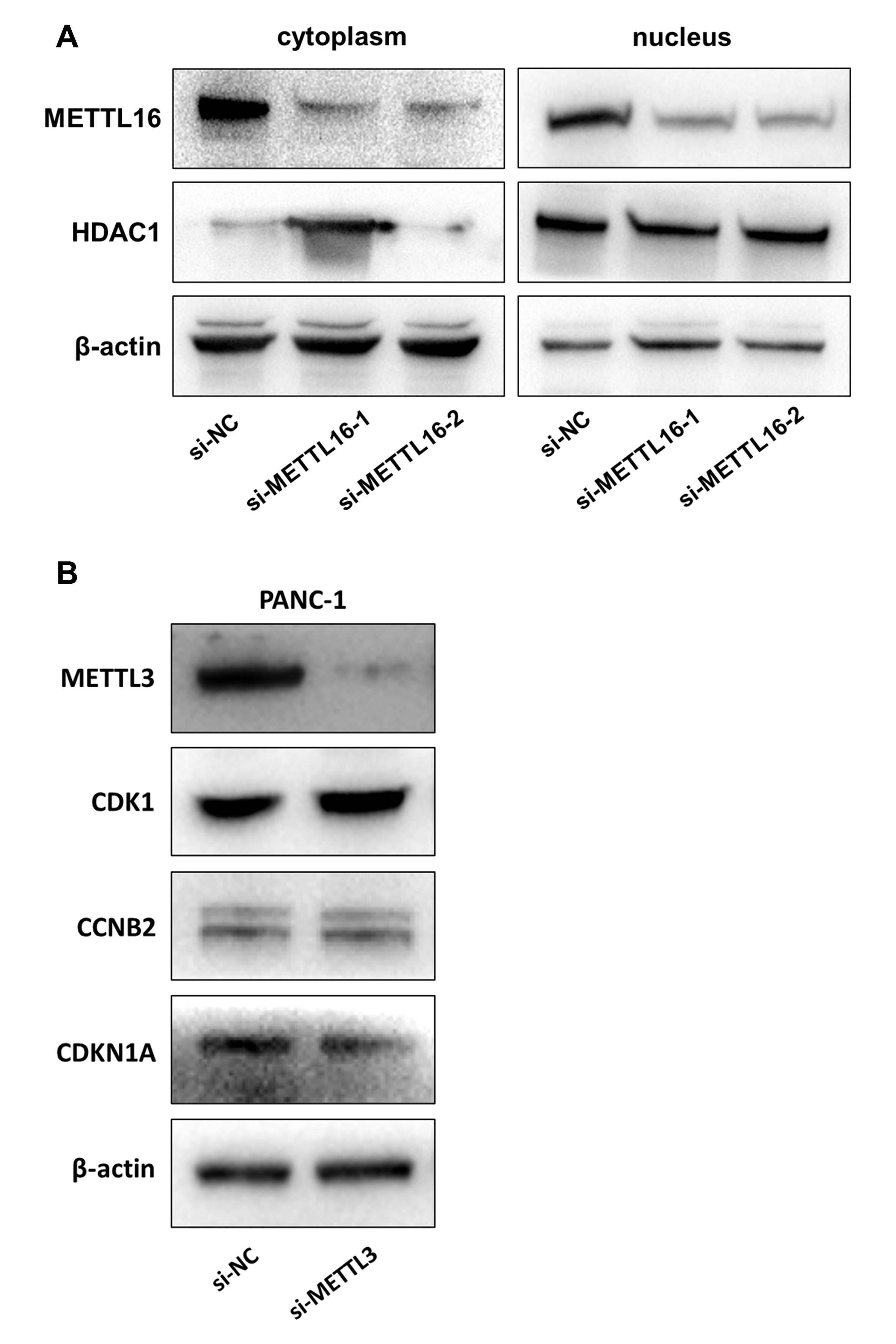

Supplement: Supplementary file 4 [file Image_4.tif]

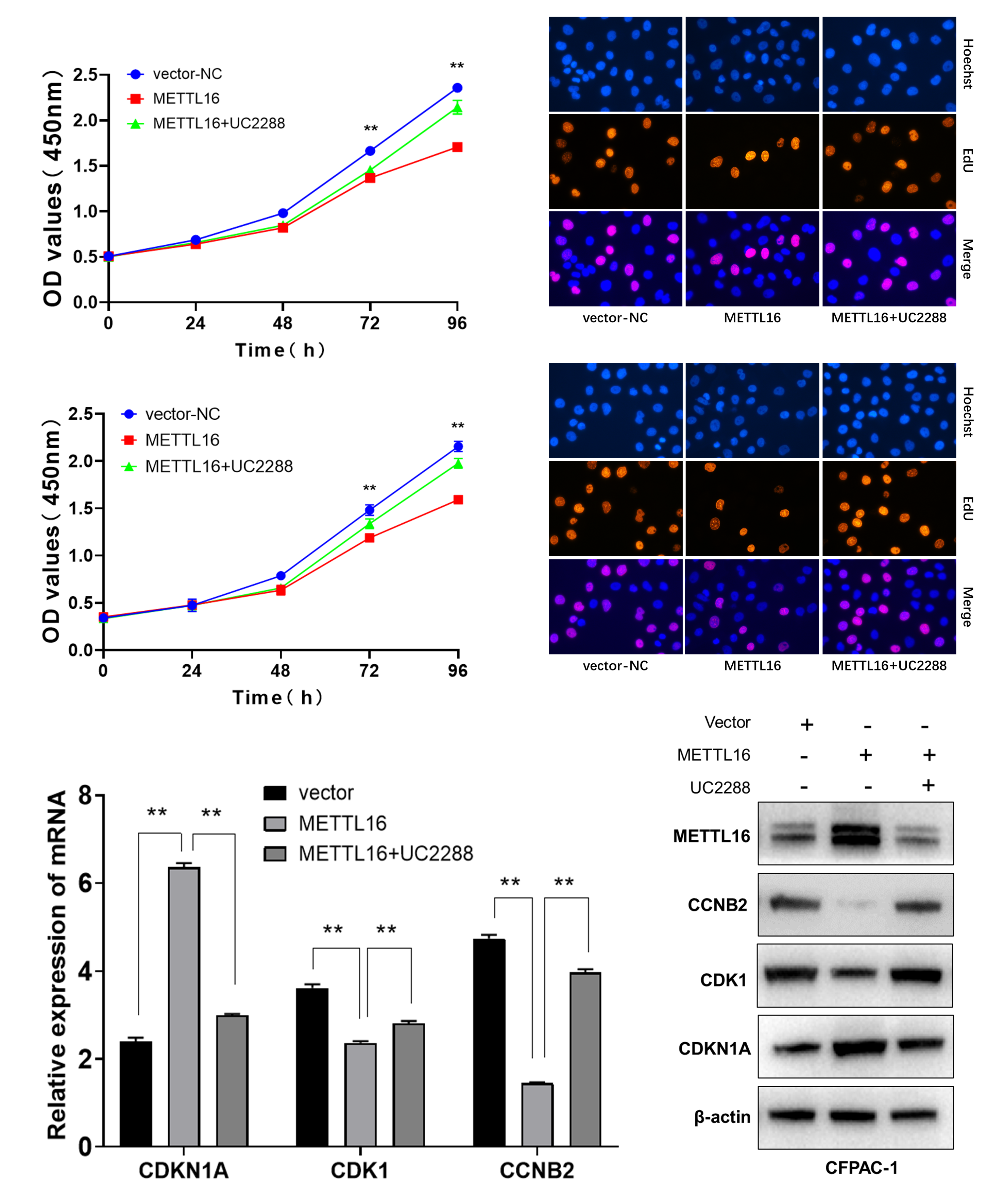

Supplement: Supplementary file 5 [file Image_5.tif]
